# Three new species of the *Stenus cirrus* group (Coleoptera, Staphylinidae) from Jiangxi, South China

**DOI:** 10.3897/zookeys.442.8215

**Published:** 2014-09-25

**Authors:** Yi-Ming Yu, Liang Tang, Wei-Dong Yu

**Affiliations:** 1 Department of Biology, Shanghai Normal University, 100 Guilin Road, 1st Educational Building 323 Room, Shanghai, 200234 P. R. China

**Keywords:** Coleoptera, Staphylinidae, *Stenus cirrus* group, China, new species, key to species

## Abstract

Three new species from Jiangxi, China, are described and illustrated: *Stenus wugongshanus* **sp. n.**, *Stenus mingyueshanus* **sp. n.**, and *Stenus songxiaobini* **sp. n.** A previously published key to the Chinese species of the *Stenus cirrus* group is modified to accommodate the new species.

## Introduction

The speciose *Stenus cirrus* group currently holds 63 species worldwide, 46 of them are known to occur in China and two in Jiangxi. Members of the group are characterized by the presence of long and erect setae on the abdomen. A detailed definition of the group was given in Tang et al. (2008) and Puthz (2009).

Among the specimens we recently collected in the Luoxiao Mountains, Jiangxi Province, three species are recognized as new and are described herein.

## Material and methods

For examination of the male genitalia, the apical three abdominal segments were detached from the body after softening in hot water. The aedeagi, together with other dissected parts, were mounted in Euparal (Chroma Gesellschaft Schmidt, Koengen, Germany) on plastic slides. Photos of sexual characters were taken with a Canon G9 camera attached to an Olympus CX31 microscope; habitus photos were taken with a Canon macro photo lens MP-E 65 mm attached to a Canon EOS7D camera.

The type specimens treated in this study are deposited in the following collections:

SHNU Department of Biology, Shanghai Normal University, P. R. China

cPut Private collection Volker Puthz, Schlitz, Germany

The measurements are abbreviated as follows:

BL body length, measured from the anterior margin of the clypeus to the posterior margin of abdominal tergite X

FL forebody length, measured from the anterior margin of the clypeus to the apicolateral angle of elytra

HW width of head including eyes

PW width of pronotum

EW width of elytra

PL length of pronotum

EL length of elytra, measured from humeral angle

SL length of elytral suture

## Taxonomy

### Stenus wugongshanus sp. n.

Taxon classificationAnimaliaColeopteraStaphylinidae

http://zoobank.org/5FFA59B8-571C-472E-A91C-66B24552A881

Figs 1 , 2 , 7–17

#### Type material.

**Holotype:** **CHINA: Jiangxi Prov.:** ♂, Pingxiang City, Wugong Shan National Park, alt. 1500–1750 m, 21.VII.2013, Song, Yin & Yu leg. (SHNU). **Paratypes:** **CHINA: Jiangxi Prov.:** 1♂, 4♀♀, same data as holotype (SHNU); 6♂♂, 3♀♀, same locality, but alt. 1000–1350 m, 20.VII.2013, Song, Yin & Yu leg. (1♂, 1♀ in cPut, rest in SHNU).

#### Description.

BL: 2.9–3.4 mm, FL: 1.4–1.7 mm.

HW: 0.66–0.77 mm, PL: 0.47–0.54 mm, PW: 0.48–0.56 mm, EL: 0.49–0.51 mm, EW: 0.54–0.63 mm, SL: 0.35–0.40 mm.

Brachypterous. Head blackish, body reddish brown, each elytron with a large ill-defined orange spot, which is about 2/3 as long and about 1/2 as broad as the respective elytron, abdomen shiny; antennae reddish yellow, club infuscate; maxillary palpi and legs reddish yellow; clypeus brown, labrum reddish brown; moderately sparsely pubescent. Paraglossae oval.

Head 1.17–1.22 times as wide as elytra; interocular area with two deep longitudinal furrows, median portion slightly convex, not reaching level of inner eye margins, with a broad impunctate line along midline; punctures round, slightly larger in median portion than near inner margins of eyes, diameter of large punctures about as wide as apex of 2nd antennal segment in cross section, interstices between punctures faintly microsculptured, narrower than half the diameter of punctures. Antennae, when reflexed, slightly extending beyond posterior margin of pronotum, antennomeres III–VIII distinctly narrower than II; IX–XI gradually broadened, forming loose club; relative length of segments from base to apex as 10.5: 6.5: 13.0: 7.5: 7.0: 5.0: 5.5: 3.5: 5.0: 6.0: 6.5.

Pronotum 0.95–1.00 times as long as wide, widest near middle and constricted at base; disk uneven, with distinct median longitudinal furrow of about half the length of pronotum and with an impunctate line along the middle; punctures round and moderately confluent, variable in size, diameter of large punctures about as wide as middle of 2nd antennal segment in cross section, interstices with dense microsculpture, much narrower than half the diameter of punctures.

Elytra 0.92–0.97 times as long as wide, distinctly constricted at base, lateral margins gradually divergent posteriad; disk uneven with distinct humeral impression, distinct sutural impression and rather faint postero-lateral impression; punctures fusiform and confluent, larger than those on pronotum, interstices between punctures microsculptured, much narrower than half the diameter of punctures.

Hind tarsi 0.70–0.75 times as long as hind tibiae, tarsomeres IV strongly bilobed.

Abdomen cylindrical; paratergites very narrow and smooth, present only in segment III, segments IV–VI with tergites and sternites entirely fused and traces of paratergites present only at base of each segment, posterior margin of tergite VII with indistinct palisade fringe; punctures round, dense at the base of each tergite, gradually becoming smaller posteriad, interstices smooth.

Male. Sternite VII with shallow emargination at middle of posterior margin, sternite VIII (Fig. 7) with semi-circular emargination at middle of posterior margin; sternite IX (Fig. 8) with long apicolateral projections, posterior margin serrate and slightly produced in the middle; tergite X (Fig. 9) with posterior margin truncate and slightly emarginated in the middle. Aedeagus (Figs 10, 11) with median lobe subparallel-sided, apical sclerotized portion triangular with round projection at apex, expulsion hooks (Fig. 13) large, strongly sclerotized; parameres slender and almost straight, extending a little beyond apex median lobe, swollen at apex, each with about 11 setae on apico-internal margin (Fig. 12).

Female. Abdomen slightly broader than that of male; sternite VIII (Fig. 14) with posterior margin weakly pointed at middle; tergite X (Fig. 15) with posterior margin truncate; spermathecal duct with basal portion strongly sclerotized and the remainder weakly sclerotized (Figs 16, 17).

**Figures 1, 2.**
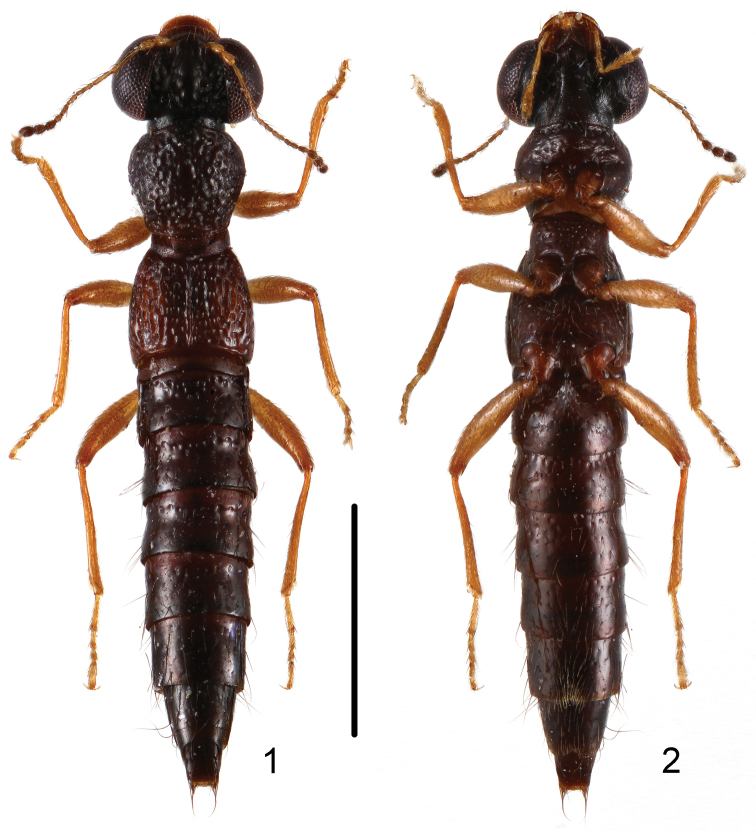
Habitus of *Stenus wugongshanus* in dorsal and ventral view. Scale bar: 1 mm.

**Figures 3, 4.**
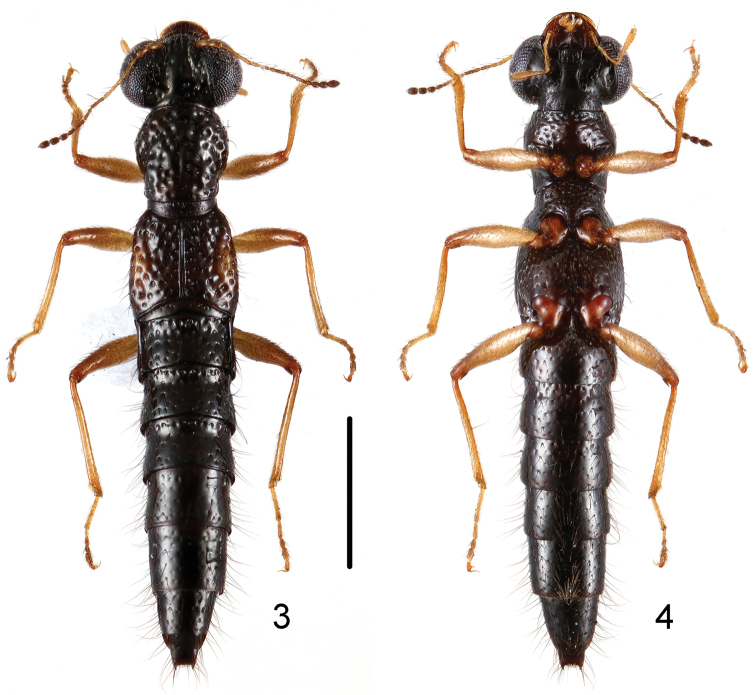
Habitus of *Stenus mingyueshanus* in dorsal and ventral view. Scale bar: 1 mm.

**Figures 5, 6.**
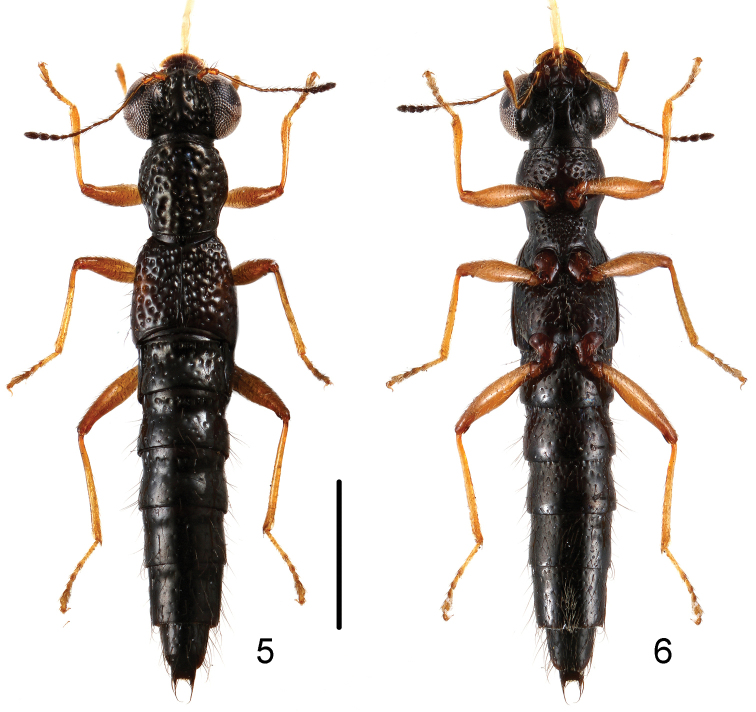
Habitus of *Stenus songxiaobini* in dorsal and ventral view. Scale bar: 1 mm.

**Figures 7–17.**
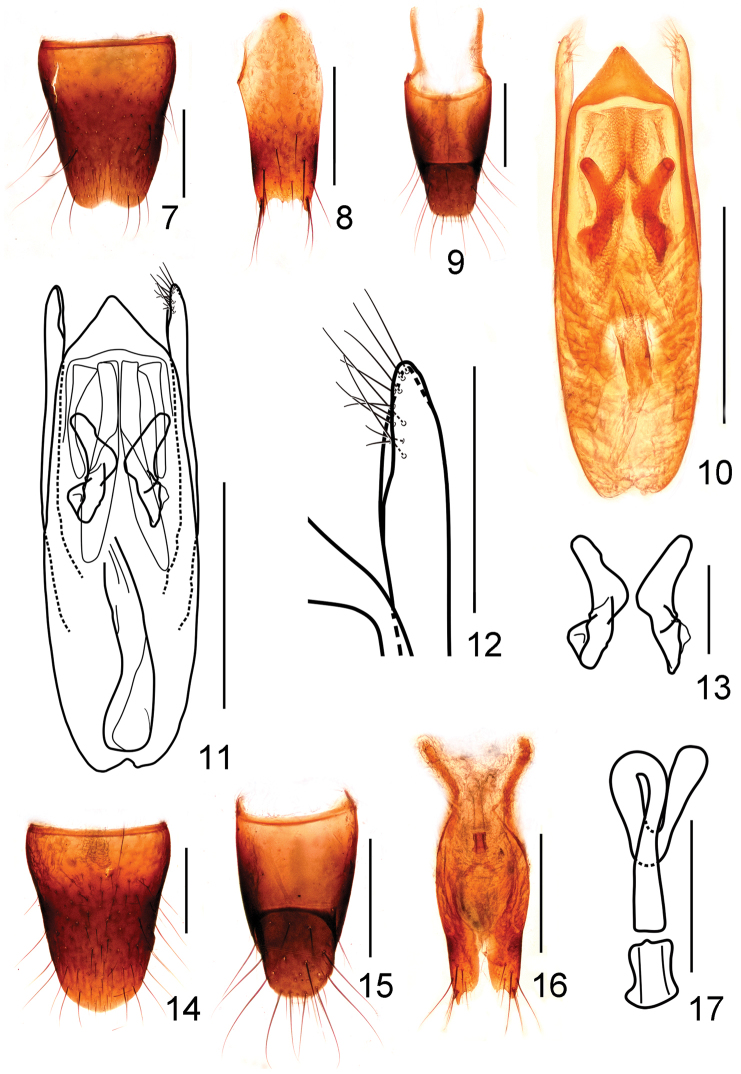
*Stenus wugongshanus*. **7** male sternite VIII **8** male sternite IX **9** male tergites IX, X **10, 11** aedeagus **12** apical portion of paramere **13** expulsion hooks **14** female sternite VIII **15** female tergites IX, X **16** valvifers and spermatheca **17** spermatheca. Scale bars: **12–13:** 0.1 mm; **7–11, 14–17:** 0.25 mm.

#### Distribution.

Jiangxi Province: Wugong Shan (China).

#### Diagnoses.

*Stenus wugongshanus* resembles *Stenus huangganmontium* Puthz, 2003 (Wuyishan, Jiangxi Province) in most aspects, but may be distinguished by the lack of paratergites on segments IV and V, which are present in *Stenus huangganmontium*.

#### Etymology.

The specific name is derived from “Wugongshan”, the type locality of this species.

### Stenus mingyueshanus sp. n.

Taxon classificationAnimaliaColeopteraStaphylinidae

http://zoobank.org/2C320675-82A8-425E-A2D7-18490431CE6F

Figs 3 , 4 , 18–27

#### Type material.

**Holotype:** **CHINA: Jiangxi Prov.:** ♂, Yichun City, Mingyue Shan, alt. 1140 m, 23.X.2013, Peng, Shen & Yan leg. (SHNU). **Paratypes:** **CHINA: Jiangxi Prov.:** 9♂♂, 5♀♀, same data as holotype (1♂, 1♀ cPut, rest in SHNU); 2♂♂, 1♀, same data but alt. 1600 m, 22.X.2013 (SHNU); 1♂, 2♀♀, Yichun City, Mingyueshan National Park, alt. 1610 m, 11.VII.2013, Song, Yin & Yu leg. (SHNU).

#### Description.

BL: 4.2–5.1 mm, FL: 2.0–2.3 mm.

HW: 0.83–0.91 mm, PL: 0.65–0.72 mm, PW: 0.63–0.69 mm, EL: 0.74–0.82 mm, EW: 0.74–0.84 mm, SL: 0.53–0.59 mm.

Brachypterous. Body blackish, each elytron with an orange marking near lateral margin, this marking about 2/3 as long and about 1/2 as broad as the respective elytron; antennae reddish yellow, club infuscate; maxillary palpi and legs reddish yellow; clypeus black, labrum brown, moderately densely pubescent. Paraglossae oval.

Head 1.08–1.12 times as wide as elytra; interocular area with two deep longitudinal furrows, median portion convex, not reaching level of inner eye margins, with a broad impunctate line along midline; punctures round to fusiform, larger and sparser in median area than near inner margins of eyes; diameter of large punctures about as wide as middle of 2nd antennal segment in cross section, interstices smooth, varying from narrower to slightly broader than half the diameter of punctures. Antennae, when reflexed, slightly extending beyond posterior margin of pronotum, antennomeres III–VIII segments distinctly narrower than II; IX–XI gradually broadened, forming loose club; relative length of segments from base to apex as 11.5: 7.5: 20.0: 10.5: 10.5: 8.5: 7.0: 5.0: 6.5: 6.0: 8.0.

Pronotum 1.01–1.04 times as long as wide, widest a little before middle and constricted at base; with shallow median longitudinal furrow of about half the length of pronotum; punctures round, some of them confluent, variable in size, diameter of large punctures much larger than middle of 2nd antennal segment in cross section, interstices smooth, distinctly narrower than half the diameter of punctures.

Elytra 0.92–1.02 times as long as wide; distinctly constricted at base, lateral margins gradually divergent posteriad; disk uneven with distinct humeral impression and faint sutural impression; punctures similar to those of pronotum, interstices smooth, narrower than half the diameter of punctures.

Hind tarsi 0.72–0.74 times as long as hind tibiae, tarsomeres IV strongly bilobed.

Abdomen cylindrical; paratergites very narrow and punctate, present only in segment III, segments IV–VI with tergites and sternites entirely fused and traces of paratergites present only at base of each segment, posterior margin of tergite VII with indistinct palisade fringe; punctures round, gradually becoming smaller posteriad, interstices smooth, varying from narrower to broader than diameter of punctures.

**Figures 18–27.**
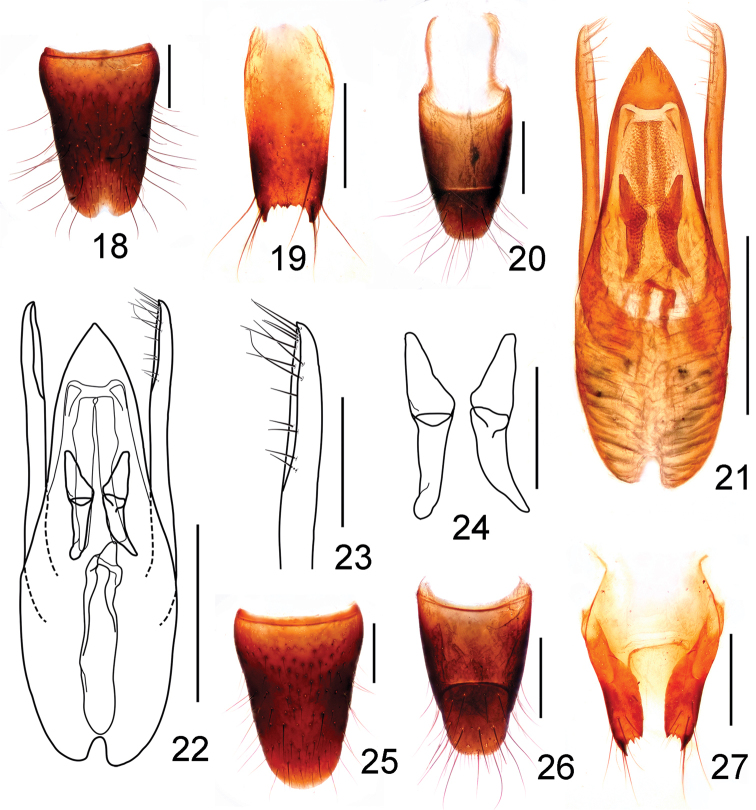
*Stenus mingyueshanus*. **18** male sternite VIII **19** male sternite IX **20** male tergites IX, X **21, 22** aedeagus **23** apical portion of paramere **24** expulsion hooks **25** female sternite VIII **26** female tergites IX, X **27** valvifers. Scale bars: **23–24:** 0.1 mm; **18–22, 25–27:** 0.25 mm.

Male. Sternite VII with shallow emargination in the middle of posterior margin, sternite VIII (Fig. 18) with triangular emargination in the middle of posterior margin; sternite IX (Fig. 19) with long and acute apicolateral projections, posterior margin serrate and nearly straight; tergite X (Fig. 20) with posterior margin convex. Aedeagus (Figs 21, 22) with median lobe subparallel-sided in basal portion and tapering in apical half, apical sclerotized portion nearly triangular, explusion hooks (Fig. 24) large, strongly sclerotized; parameres almost straight, distinctly longer than median lobe, each with about 12–13 setae on apico-internal margin (Fig. 23).

Female. Sternite VIII (Fig. 25) with posterior margin rounded; tergite X (Fig. 26) slightly emarginated at middle of posterior margin; without sclerotized spermatheca (Fig. 27).

#### Distribution.

Jiangxi Province: Mingyue Shan (China).

#### Diagnoses.

*Stenus mingyueshanus* resembles *Stenus ovalis* Tang, Li & Zhao, 2005 (Wuyanling, Zhejiang Province), but may be distinguished by the distinctly coarser and sparser punctation of the forebody in the new species.

#### Etymology.

The specific name is derived from “Mingyueshan”, the type locality of this species.

### Stenus songxiaobini sp. n.

Taxon classificationAnimaliaColeopteraStaphylinidae

http://zoobank.org/4B067492-9B22-4426-8B8E-99D7956193CD

Figs 5 , 6 , 28–37

#### Type material.

**Holotype:** **CHINA: Jiangxi Prov.:** ♂, Pingxiang City, Wugong Shan National Park, alt. 1340–1400 m, 19.VII. 2013, Song, Yin & Yu leg. (SHNU). **Paratypes:** **CHINA: Jiangxi Prov.:** 1♂, 1♀, same data, but alt. 1000–1350 m, 20.VII.2013 (SHNU).

#### Description.

BL: 4.3–4.7 mm, FL: 2.0–2.1 mm.

HW: 0.82–0.83 mm, PL: 0.62–0.67 mm, PW: 0.63–0.64 mm, EL: 0.74–0.78 mm, EW: 0.73–0.76 mm, SL: 0.52–0.57 mm.

The new species is similar to *Stenus mingyueshanus* sp. n. in most respects, but different in the following characters: head 1.08–1.12 times as wide as elytra; frons with interstices smooth, varying from narrower to slightly broader than half the diameter of punctures; relative length of antennal segments from base to apex as 9.5: 7.0: 18.5: 10.5: 8.5: 8.0: 6.5: 4.0: 6.0: 6.0: 7.0.

Pronotum 1.08–1.13 times as long as wide; median longitudinal furrow shallow of about 2/5 the length of pronotum, punctures round and partly confluent, variable in size, diameter of large punctures slightly larger than middle of 2nd antennal segment in cross section; interstices smooth, distinctly narrower than half the diameter of punctures.

Elytra 1.02–1.03 times as long as wide; size of punctures similar to those of pronotum, interstices partly with faint reticulation, somewhat narrower than half the diameter of punctures.

Hind tarsi 0.77–0.80 times as long as hind tibiae.

**Figures 28–37.**
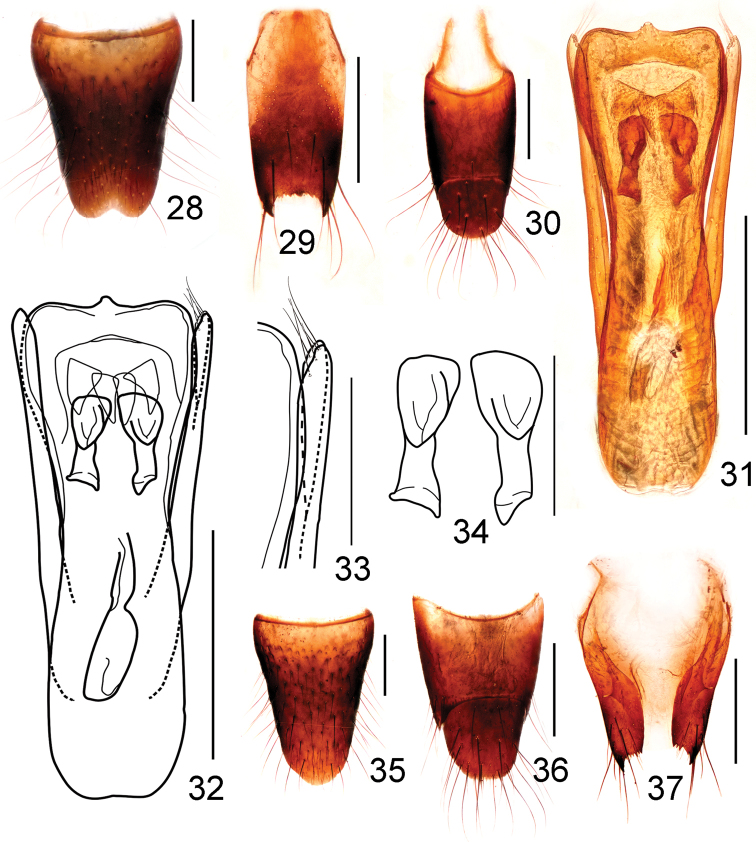
*Stenus songxiaobini*. **28** male sternite VIII **29** male sternite IX **30** male tergites IX, X **31, 32** aedeagus **33** apical portion of paramere **34** expulsion hooks **35** female sternite VIII **36** female tergites IX, X **37** valvifers. Scale bars: **33–34:** 0.1 mm; **28–32, 35–37:** 0.25 mm.

Male. Sternite VII with shallow emargination in the middle of posterior margin, sternite VIII (Fig. 28) with a circular emargination in the middle of posterior margin; sternite IX (Fig. 29) with long and acute apicolateral projections, posterior margin serrate and nearly straight; tergite X (Fig. 30) with posterior margin broadly rounded. Aedeagus (Figs 31, 32) with median lobe subparallel-sided in basal portion and broadening in apical half, apical margin truncate with a median projection, explusion hooks (Fig. 34) large, strongly sclerotized; parameres almost straight, extending a little beyond apex of median lobe, each with about 5 setae on apico-internal margin (Fig. 33).

Female. Sternite VIII (Fig. 35) with convex posterior margin; tergite X (Fig. 36) with posterior margin convex; spermatheca unsclerotized (Fig. 37).

#### Distribution.

Jiangxi Province: Wugong Shan (China).

#### Diagnoses.

*Stenus songxiaobini* is similar to *Stenus mingyueshanus* sp. n., but may be distinguished from the latter by denser and deeper punctures on the pronotum and elytra and by the shallower median longitudinal furrow, which is about 2/5 the length of pronotum.

#### Etymology.

This species is named in honor of Mr. Xiao-Bin Song, collector of the new species.

### Modified couplets of the key (Pan et al. 2012) to Chinese species of the *Stenus cirrus* group

**Table:** 

10a	Pronotum with interstices distinctly reticulated. Habitus: Figs 1, 2; sexual characters: Figs 7–17. BL: 2.9–3.4 mm. China: Jiangxi: Wugongshan	*Stenus wugongshanus* sp. n.
–	Pronotum with interstices smooth	10b
10b	Head narrower than elytra or nearly as wide as elytra	11
–	Head distinctly wider than elytra	13
18a	Larger species, FL ≥ 1.9 mm; elytral markings longer than half the length of elytra, extending towards humeral angles	18b
–	Smaller species, FL = 1.6–1.7 mm, elytral markings shorter than half the length of elytra, not extending towards humeral angles. Habitus: figure 1 in Tang et al. (2005); sexual characters: figures 4–7 in Tang et al. (2005). BL: 3.2–4.1 mm. China: Hubei: Houhe	*Stenus andoi* Tang, Li & Zhao, 2005
18b	Punctures on pronotum very large, diameter of large punctures much larger than middle of 2nd antennal segment in cross section. Habitus: Figs 3–4; sexual characters: 18–27. BL: 4.2–5.1 mm. China: Jiangxi: Mingyueshan	*Stenus mingyueshanus* sp. n.
–	Punctures on pronotum relatively small, diameter of large punctures slightly larger than middle of 2nd antennal segment in cross section	18c
18c	Pronotum without median longitudinal furrow; punctation of abdominal tergites coarser, punctures on abdominal tergite IV as large as those of elytra. Habitus: figure 3 in Tang, Li & Zhao (2005); sexual characters: figures 12–15 in Tang et al. (2005). BL: 3.8–4.5 mm. China: Zhejiang: Wuyanling	*Stenus ovalis* Tang, Li & Zhao, 2005
–	Pronotum with shallow median longitudinal furrow; punctation of abdominal tergites less coarse, punctures on abdominal tergite IV smaller than those of elytra. Habitus: Figs 5, 6; sexual characters: Figs 28–37. BL: 4.3–4.7 mm. China: Jiangxi: Wugongshan	*Stenus songxiaobini* sp. n.

## Supplementary Material

XML Treatment for Stenus wugongshanus

XML Treatment for Stenus mingyueshanus

XML Treatment for Stenus songxiaobini

## References

[B1] Pan Y-H Tang L Li L-Z (2012) Notes on the *Stenus cirrus* group, with description of two new species from China (Coleoptera, Staphylinidae). Zookeys 169: 61–77. doi: 10.3897/zookeys.169.2647 22371686 10.3897/zookeys.169.2647 PMC3278815

[B2] Puthz V (2003) Neue und alte Arten der Gattung *Stenus* Latreille aus China (Insecta: Coleoptera: Staphylinidae: Steninae). Entomologische Abhandlungen 60: 148–149

[B3] Puthz V (2009) The group of *Stenus cirrus* in Taiwan (Coleoptera: Staphylinidae) (310^th^ Contribution to the Knowledge of Steninae). Entomological Review of Japan 64(2): 115–133

[B4] Tang L Li L-Z Zhao M-J (2005) Three new species of the group of *Stenus cirrus* (Coleoptera, Staphylinidae) from China. Elytra, Tokyo 33(2): 609–616

[B5] Tang L Zhao Y-L Puthz V (2008) Six new *Stenus* species of the *cirrus* group (Coleoptera, Staphylinidae) from China with a key to species of the group. Zootaxa 1745: 1–18

